# Psychological status on informal carers for stroke survivors at various phases: a cohort study in China

**DOI:** 10.3389/fpsyt.2023.1173062

**Published:** 2023-05-30

**Authors:** Neng Huang, Yidan Tang, Peng Zeng, Xingtong Guo, Zuoyan Liu

**Affiliations:** ^1^Department of Rehabilitation Medicine Center, West China Hospital, Sichuan University/West China School of Nursing, Sichuan University, Chengdu, China; ^2^Key Laboratory of Rehabilitation Medicine in Sichuan Province, Chengdu, Sichuan, China; ^3^School of Nursing, Chengdu University of Traditional Chinese Medicine, Chengdu, China

**Keywords:** stroke survivors, informal carers, psychology, influence factors, cohort study

## Abstract

**Background:**

In China, the risk of stroke is higher than that in developed countries such as Europe and North America. Informal caregivers play a major role in providing support to stroke survivors. Currently, only limited studies on changes in psychological state of the caregivers at different stages of stroke have been published.

**Purpose:**

To investigate the stress and psychological state of informal caregivers of stroke patients in different periods and to explore the factors that affect their states.

**Methods:**

202 informal caregivers of stroke patients were selected in a 3a-grade hospital in Chengdu, Sichuan. Follow-up was conducted by face-to-face interviews, telephone calls, or home visits on days 3, 2 months, and 1 year after onset. We investigated the basic information about the caregivers, including their anxiety, depression and social support conditions. We analyzed the pressure and psychological conditions of informal caregivers at different stages of stroke and analyzed its influencing factors. The data were displayed by the number and percentage of the cases; the continuous variables were described by means and standard deviation. In addition, the data were compared by Pearson correlation analysis and logistic regression analysis.

**Results:**

(1) Within 3 days after the onset of stroke, the informal caregivers had the highest stress, the most severe anxiety and depression, the heaviest burden, and the lowest score of medical-social support. Over time, the pressure and burden of the caregivers are gradually decreasing, anxiety and depression are increasing, and social support is also increasing. (2) The stress and psychological status of informal stroke caregivers are affected by multiple factors, including the caregiver’s age, relationship with the patient, patient’s age, and patient’s physical conditions.

**Conclusion:**

The stress and psychological status of informal caregivers varied in the different stages of stroke, and they were affected by several factors. Medical staff should pay attention to informal caregivers while providing care for patients. Relevant interventions may be developed based on the results to improve the health of informal caregivers and thus to promote the health of patients.

## 1. Background

Globally, stroke is the second leading cause of death and the third most important cause of disability burden (1, 2). The cost of stroke is high, estimated $33 billion (including the expenses of health care, medicines and missed work) in the United States, $ 10.9 billion *per annum* in the United Kingdom, and $5 billion in Australia (3). In China, the risk of stroke is much higher than that in developed countries such as Europe and America (4). Cerebrovascular disease has jumped to the top of the causes of death (5) and is also the main cause of long-term disability in adults (6). In China, the annual social and economic burden caused by stroke has exceeded $5.8 billion (7).

During the hospitalization of stroke patients, their primary caregivers are prone to negative coping styles and interpersonal relationships, and the incidence of caregiving burden among primary caregivers of stroke patients ranges from 25 to 54% (8, 9). In our study, more than 80% of caregivers had caregiving burdens, 6.7% had severe caregiving burdens, and 72.0% had mild to moderate caregiving burden scores (10). Other studies have shown that more than 60% of caregivers have a moderate or higher caregiving burden score (11).

Informal caregivers are different from formal caregivers who have some degree of training and are paid for their service (12, 13). The informal caregivers are usually family members, friends and acquaintances who have a close relationship with the patients (14, 15). China is a country that is called “has not rich but old,” and the pension mechanism and medical and health security system are still in construction. Therefore, informal caregivers play a major role in providing support to stroke survivors.

The lack of available stroke information and caregiving training (16, 17), lack of attention for healthcare providers (18), and lack of time to engage in social activities (12) result in a variety of psychiatric symptoms among caregivers, such as stress, anxiety and depression. These psychiatric symptoms may have potential negative impacts on caregivers (19, 20). Caregivers perceived stress to be higher than that of other populations, and stress levels were consistent across 6 weeks poststroke (21, 22). Worldwide, the prevalence of depressive symptoms in caregivers of stroke survivors is approximately 35%, almost twice as high as that in the general population and the aggregated prevalence of anxiety symptoms is 21.4% (19). In Africa, caregivers are often physically and emotionally burdened by the limited support available to stroke caregivers and the limited care provided to patients due to hospital medical conditions (23). A study conducted by Indian scholars on primary caregivers of stroke patients showed that 80% of those who rated themselves as financially overburdened, 76 and 43% of caregivers who experienced depression and anxiety and sleep dysfunction, respectively, and 70% of those who felt overloaded with caregiving workload (24). In the United States, in a correlation analysis of health-related quality of life among African American and Caucasian stroke caregivers by Clay et al. (25) both objective stressors and psychological well-being influenced caregiver quality of life.

Poststroke patients will experience three stages, including the acute stage (≤ 72 h), subacute stage (72 h–3 months) and chronic stage (≥ 3 months) (26). At present, there is a lack of studies with follow-up surveys on the stress and psychological status of informal caregivers of stroke patients in China. In addition, there are also only limited studies on the stress and psychological changes of caregivers in different stages of stroke patients. Therefore, the aim of this study was to understand the stress and psychological status of informal caregivers at different stroke stages and probe the related factors in the Chinese context to provide a reference for later intervention.

## 2. Methods

### 2.1. Study design and participants

In this study, the method of convenience sampling was adopted to select 252 informal stroke caregivers. The stroke center of a third-grade first-class hospital in China from October 2016 to September 2018 as the research objects. Face-to-face interviews were conducted within 3 days after the onset of the disease by same one, and in-home or telephone follow-up was conducted 2 months and 1 year after the onset. The interviewer listed fixed questions before the interview to ensure that each interviewee was interviewed homogeneously. Inclusion criteria included (1) caregivers of stroke patients confirmed by head CT scan; (2) caregivers for patients without cognitive impairment. The exclusion criteria were as follows: (1) the age of the caregiver was less than 18 years old; (2) patients died or were lost to follow-up during the study; and (3) caregivers refused to cooperate with the investigation. During the investigation, 4 patients died, 17 caregivers were rejected the caregiving process changed to other caregivers, 29 caregivers were lost to follow-up between May 2017 and March 2018 due to factors such as a change in the caregiver’s phone number, and 202 caregivers were finally included.

### 2.2. Measures

#### 2.2.1. Outcome variables

The stress of informal caregivers was assessed using the Chinese version of the Perceived Stress Scale (PSS) (27). It includes 14 items using the Likert 5-level scoring method. Each question has 0 to 4 points, and the total score of the scale is evaluated. Scores of 0–28 points, 29–42 points, and 43–56 points indicate that stress is not threatening health, stress might threatens health, and stress may seriously threaten health, respectively.

Informal caregivers’ anxiety was assessed with the self-rating anxiety scale (SAS), which consists of 20 items. It uses a 4-level scoring method to assess the frequency of symptoms. A high total score indicates a high degree of anxiety (28). According to the results of the Chinese norm, the SAS standard is divided into 50 points, of which 50–59 are classified as mild anxiety, 60–69 are classified as moderate anxiety, and 70 to above are classified as severe anxiety (28, 29).

Informal caregivers’ depression was assessed with a self-rating depression scale (SDS). The scale has a total of 20 items, using a four-level scoring method, adding the scores of the 20 items, that is, obtaining the rough points; multiplying the coarse points by 1.25 and taking the whole part to obtain the standard score, a higher standard score indicates more severe depression. According to the Chinese norm results, the SDS standard is divided into three levels: 53–62 classified as mild depression, 63–72 classified as moderate depression, and ≥ 72 classified as severe depression (29).

We used the Chinese version of the Zarit Caregiver Burden Interview (ZBI) to assess the burden of informal caregivers (30). It contains 22 items, and response options range from 0 to 4. The total score ranges from 0 to 88. The total score between 21 and 40 indicates no burden or mild burden, a score between 41 and 60 represents moderate, while a severe burden exists in people with a ZBI score of 61 or more (31).

We evaluated informal caregivers’ degree of social support using the Chinese version of the Medical Outcome Study Social Support Survey (MOS-SSS) (32). The MOS-SSS is a brief, multidimensional, self-administered, social support survey that was developed to assess 1 item for functional social support and four dimensions: emotional/informational, tangible, affectionate, and positive social interaction. The MOS-SSS consists of 19 items rated on a 5-point Likert-type scale, and we converted the sums of the scores into values from 0 to 100. We defined high social support as an MOS-SSS-C score above the mean of the sample.

#### 2.2.2. Independent variables

##### 2.2.2.1. Characteristics of informal caregivers

Data on age, gender, education level and poststroke working status were collected. The educational level was classified according to the standard Chinese classification system and then dichotomized into low and high education, the latter including college education and higher. Prestroke working status was divided into “employment” and “retirement or no employment.” The relationship between the caregiver and the patient included spouses and children, relatives, friends and other relationships.

##### 2.2.2.2. Patient characteristics

Data on age and sex were obtained from the patients. The Modified Barthel Index (MBI) was used to assess the patients’ independence in activities of daily living (ADL). This instrument is valid and reliable in stroke populations (33). The Chinese version of the Instrumental activities of daily living scale (IADL) was used to measure Advanced daily life activity. The evaluation of IADL can reflect the ability of patients to return to life and society (34). The Modified Rivermead Mobility Index (MRMI) was used to assess the mobility of patients after stroke. The tasks involved in the MRMI assessment are simple, functional, and essential (35). Stroke severity was measured using the National Institutes of Health Stroke Scale (NIHSS) 4 days poststroke (36). The patient’s anxiety and depression assessment tools were consistent with the caregiver’s assessment tool.

### 2.3. Statistical analyses

Data analysis was performed using SPSS 22.0. The data were displayed by the number of and percentage of the cases; the continuous variables were described by means and standard deviation. In addition, the data were compared by Pearson correlation analysis and logistic regression analysis, and *p* < 0.05 (two-sided) was considered statistically significant.

## 3. Results

### 3.1. Baseline characteristics

A total of 252 stroke survivors were included. If the patient dropped out, the caregivers was excluded. Four patients died during follow-up, 17 caregivers were rejected due to the caregiving process changed to other caregivers, 29 caregivers were lost to follow-up between May 2017 and March 2018 due to factors such as a change in the caregiver’s phone number and 202 caregivers were eventually enrolled. The average age of stoke patients was 54.28 ± 13.33 years old, including 116 males (57.43%). The mean and SD of the MBI score was 50.77 ± 16.72, which indicates moderate dysfunction in the daily life activities of stroke survivors. The IADL score was 7.23 ± 5.07, indicating that the patient’s instrumental daily living ability was slightly impaired. The NIHSS score was 6.56 ± 3.41, indicating that most of the patients had moderate stroke. The SAS and SDS scores were 40.90 ± 10.75 and 41.10 ± 9.28, respectively, and the patient had no obvious anxiety or depression. A total of 202 informal caregivers of stroke patients were enrolled in this survey, including 48 males and 154 females, with an average age of 51.01 ± 12.50 years; 69.31% were spouse relationships, and 30.69% were other relationships; only 43.56% of caregivers had a full-time job when participating in the survey; caregivers were mainly low-educated, accounting for 56.44% (see Table 1).

**Table 1:** Informal caregiver and patient characteristics (*N* = 202).

Characteristics	Mean (SD)	%
Informal caregivers’ characteristics		
age	51.01 ± 12.50	
Gender		
Male	48	23.76
Female	154	76.24
Education level		
Low education	114	56.44
High education	88	43.56
Prestroke working status		
Employment	88	43.56
Retirement or no employment	114	56.44
The relationship between caregivers and patients		
Spouse	140	69.31
Other relationships	62	30.69
Patients characteristics		
Age	54.28 ± 13.33	
Gender		
Male	116	57.43
Female	86	42.57
MBI	50.77 ± 16.72	
IADL	7.23 ± 5.07	
MRMI	22.54 ± 9.05	
NIHSS	6.56 ± 3.41	
SAS	40.90 ± 10.75	
SDS	41.10 ± 9.28	
Type of stroke		
Intracerebral hemorrhage	95	47.03
Ischemic stroke	86	42.57
Subarachnoid hemorrhage	13	6.44
Stroke of undetermined type	8	3.96
Comorbidities		
Yes	193	95.54
No	9	4.46

### 3.2. Informal caregiver outcomes at different stages

Through the different stages of post-stroke rehabilitation, refined and targeted health education is developed according to the patient’s condition and informal caregivers, such as knowledge of the disease, rehabilitation training, knowledge of medication, observation of the condition, psychological support, home environment modification and other knowledge education. The study found that the perceived stress of informal caregivers was the highest in the acute stage of the patient (within 72 h after stroke onset), reaching 41.17 ± 1.877 points. After entering the subacute stage and the chronic stage (the survey time was 2 months and 1year after the onset of stroke), the perceived stress significantly decreased to 30.84 ± 6.400 and 16.69 ± 4.462, respectively, and the stress was within the normal range. Additional information is detailed in Table 2.

**Table 2:** Informal caregiver outcomes at different stages (scores).

	Stages of poststroke			*p* value
	3 days	2 months	1 year	
PSS	41.17 ± 1.877	30.84 ± 6.400	16.69 ± 4.462	< 0.05
Anxiety (SAS)	48.62 ± 2.258	34.94 ± 5.043	27.35 ± 2.590	< 0.05
Depression (SDS)	55.08 ± 2.622	40.95 ± 6.062	30.23 ± 3.101	< 0.05
ZBI	62.04 ± 3.589	34.63 ± 7.828	28.01 ± 5.369	< 0.05
MOS-SSS	69.09 ± 11.13	74.00 ± 13.12	75.76 ± 13.14	< 0.05

The results in Table 3 show that there was a certain trend in informal stroke caregivers’ stress. Over time, the perceived stress of caregivers improved, the number of no threats to health increased, and the number of threats to health and serious threats to health decreased. Only 38.61% of the informal caregivers showed anxiety within 3 days of onset, but 90.10% of them showed depression, which decreased after 2 months. Over time, the burden on caregivers gradually diminished, but 7.9 percent of caregivers still felt a moderate burden.

**Table 3:** Informal caregiver outcomes at different stages.

Outcomes	Category	Stages of poststroke			Test statistic	*p* value
		3 days (*n*, %)	2 months (*n*, %)	1 year (*n*, %)		
PSS	No threaten to health (0–28)	0 (0)	82 (40.59)	141 (69.8)	431.22	< 0.001
	Might threaten to health (29–42)	186 (92.08)	112 (55.45)	61 (30.2)	233.17	
	Seriously threaten to health (43–56)	16 (7.92)	8 (3.96)	0	168.83	
Anxiety (SAS)	No (< 50)	124 (61.39)	202 (100)	202 (100)	324.52	
	Mild (50 ~ 59)	68 (38.61)	0	0	96.5	< 0.001
	Moderate (60 ~ 69)	0	0	0	390.62	
	Severe (≥ 70)	0	0	0	103.72	
Depression(SDS)	No (< 53)	20 (9.90)	200 (99.01)	202 (100)	101.5	
	Mild (53–62)	180 (89.11)	2 (0.99)	0	413.28	< 0.001
	Moderate (63–72)	2 (0.99)	0	0	349.56	
	Severe (> 73)	0	0	0	239.6	
ZBI	No burden (< 20)	0	10 (4.95)	26 (12.9)	101.5	< 0.001
	Mild burden (20 ~ 39)	0	138 (68.32)	160 (79.2)	431.22	
	Moderate burden (40 ~ 59)	50 (24.75)	54 (26.73)	16 (7.9)	233.17	
	Serve burden (≥60)	152 (75.25)	0	0	168.83	
MOS-SSS		69.09 ± 11.13	74.00 ± 13.12	75.76 ± 13.14	15.450	< 0.001

### 3.3. Analysis of related factors of different evaluation indicators of caregivers

Pearson correlation analysis showed that the factors affecting the stress in informal caregivers included the caregiver’s own occupational status, relationship with the patient, and medical-social support. In addition, the patient’s functional status, such as ADL, anxiety, NHISS score, etc., also had an impact on caregiver stress (see Table 4).

**Table 4:** Factors affecting the pressure level of caregivers.

	3 days		2 months		1 year	
	*r*	*p* value	*r*	*p* value	*r*	*p* value
Informal caregivers						
Age	−0.0130	0.865	−0.0030	0.753	−0.0010	0.698
Occupation status	0.9760	0.000	0.9850	0.001	0.9640	0.000
Education level	−0.0070	0.056	−0.0040	0.062	−0.0020	0.112
Relationship with patients	0.9752	0.014	0.9653	0.002	0.9867	0.000
MOS-SSS	0.9621	0.015	0.9681	0.008	0.9784	0.003
Patients						
Gender	−0.0080	0.846	−0.0060	0.823	−0.0020	0.815
Age	0.0030	0.859	0.0090	0.798	0.0130	0.782
MBI	0.9665	0.001	0.9813	0.000	0.9613	0.001
IADL	0.9973	0.010	0.9925	0.002	0.9861	0.003
MRMI	0.9693	0.003	0.9816	0.011	0.9717	0.010
SAS	0.9871	0.000	0.9753	0.000	0.9983	0.000
SDS	0.9913	0.000	0.9845	0.013	0.9653	0.001
NHISS	0.9812	0.000	0.9937	0.023	0.9561	0.000

Caregivers’ age, occupational status, relationship with patients, and medical-social support could also affect the anxiety of informal caregivers, while influencing factors related to patients included patients’ age, MBI scores, and MHISS scores (see Table 5).

**Table 5:** Factors affecting the anxiety of caregivers.

	3 days		2 months		1 year	
	*r*	*p* value	*r*	*p* value	*r*	*p* value
Informal caregivers						
Age	0.9581	0.004	0.9581	0.000	0.9717	0.000
Occupational status	0.9861	0.000	0.9861	0.000	0.9983	0.000
Education level	0.0390	0.379	−0.0390	0.358	−0.0020	0.456
Relationship with patients	0.9760	0.000	0.9760	0.002	0.9613	0.000
MOS-SSS	0.9841	0.018	0.9841	0.011	0.9861	0.009
Patients						
Gender	0.0340	0.789	0.0340	0.756	0.0340	0.729
Age	0.9813	0.001	0.9813	0.001	0.9813	0.001
MBI	0.9703	0.023	0.9703	0.015	0.9703	0.008
IADL	0.0130	0.235	−0.0130	0.221	−0.0130	0.198
MRMI	0.0078	0.569	0.0078	0.554	0.0078	0.456
SAS	0.0095	0.456	0.0095	0.322	0.0095	0.256
SDS	0.0071	0.528	0.0071	0.489	0.0071	0.563
NHISS	0.9913	0.001	0.9913	0.002	0.9913	0.000

Consistent with the influencing factors of anxiety, the depression of informal caregivers was also affected by caregivers’ age, relationship with patients and medical-social support. Meanwhile, the age, MBI score and NHISS score of the patients also affected the caregiver’s depression level (see Table 6) (Tables 7–11).

**Table 6:** Factors affecting the depression of caregivers.

	3 days		2 months		1 year	
	*r*	*p* value	*r*	*p* value	*r*	*p* value
Informal caregivers						
Age	0.9765	0.000	0.9765	0.000	0.9765	0.000
Occupational status	−0.0076	0.069	−0.0076	0.092	−0.0076	0.118
Education degree	−0.008	0.072	−0.008	0.086	−0.008	0.126
Relationship with patients	0.9954	0.018	0.9954	0.007	0.9954	0.000
MOS-SSS	0.9913	0.000	0.9913	0.002	0.9913	0.009
Patients						
Gender	−0.013	0.785	−0.0151	0.726	−0.013	0.699
Age	0.9973	0.006	0.9954	0.000	0.9967	0.000
MBI	0.9651	0.003	0.9618	0.000	0.9601	0.000
IADL	−0.0351	0.145	−0.0135	0.844	−0.0312	0.356
MRMI	−0.0421	0.133	−0.0123	0.218	−0.0425	0.253
SAS	−0.0131	0.313	−0.0164	0.433	−0.0135	0.396
SDS	−0.0156	0.370	−0.0171	0.396	−0.0165	0.412
NHISS	0.9813	0.000	0.9825	0.000	0.9830	0.000

**Table 7:** Multivariate stepwise regression analysis of factors influencing caregiver perceived stress status.

Influence factors	Regression coefficient	Standard error	Standard regression coefficient	*T* value	*p* value
informal caregivers					
Age	2.088	0.373	0.285	5.453	0.000
Occupational status	−0.839	0.301	−0.171	−3.267	0.001
Education degree	2.158	0.600	0.235	3.605	0.000
Relationship with patients	−3.799	0.743	−0.402	−5.124	0.000
MOS-SSS	3.241	0.779	0.303	4.140	0.000
Patients					
Gender	−0.307	0.146	−0.111	−2.115	0.037
Age	0.101	0.038	0.157	2.763	0.005
MBI	0.080	0.037	0.115	2.061	0.039
IADL	0.105	0.050	0.126	2.258	0.024
MRMI	−0.391	0.111	−0.372	−3.556	0.000
SAS	0.223	0.092	0.234	2.461	0.015
SDS	0.265	0.123	0.198	2.203	0.027
NHISS	0.033	0.006	0.260	4.741	0.000

**Table 8:** Multivariate stepwise regression analysis of the influencing factors of caregiver anxiety symptoms.

Influence factors	Regression coefficient	Standard error	Standard regression coefficient	*T* value	*p* value
Informal caregivers					
Age	3.055	0.881	0.227	3.451	0.001
Occupational status	−2.430	0.811	−0.166	−3.015	0.003
Education degree	−2.685	0.843	−0.175	−3.200	0.002
Relationship with patients	1.150	0.445	0.145	2.710	0.006
MOS-SSS	−0.768	0.358	−0.113	−2.153	0.033
Patients					
Gender	0.922	0.434	0.110	2.079	0.034
Age	−2.421	0.915	−0.203	−2.635	0.009
MBI	2.645	1.340	0.175	1.921	0.055
IADL	0.685	0.384	0.089	1.795	0.072
MRMI	0.155	0.043	0.214	3.771	0.000
SAS	0.107	0.035	0.159	2.768	0.005
SDS	0.095	0.045	0.120	2.096	0.037
NHISS	−0.085	0.039	−0.119	−2.173	0.030

**Table 9:** Multivariate stepwise regression analysis of factors influencing symptoms of depression in caregivers.

Influence factors	Regression coefficient	Standard error	Standard regression coefficient	*T* value	*p* value
Informal caregivers					
Age	0.095	0.045	0.113	2.029	0.041
Occupational status	0.027	0.006	0.236	4.247	0.000
Education degree	0.109	0.045	0.137	2.489	0.013
Relationship with patients	0.221	0.081	0.268	2.709	0.006
MOS-SSS	0.030	0.006	0.250	4.820	0.000
Patients					
Gender	−0.305	0.091	−0.257	−3.151	0.002
Age	0.220	0.075	0.284	2.819	0.005
MBI	−0.131	0.060	−0.110	−2.149	0.031
IADL	−0.033	0.014	−0.117	−2.289	0.022
MRMI	0.111	0.041	0.138	2.515	0.011
SAS	−0.147	0.075	−0.137	−1.959	0.047
SDS	0.456	0.091	0.485	4.874	0.000
NHISS	0.335	0.097	0.357	3.441	0.001

**Table 10:** Multivariate stepwise regression analysis of factors influencing caregiver burden.

Influence factors	Regression coefficient	Standard error	Standard regression coefficient	*T* value	*p* value
Informal caregivers					
Age	−3.787	0.740	−0.401	−5.130	0.000
Occupational status	3.243	0.781	0.305	4.143	0.000
Education degree	−0.151	0.049	−0.121	−2.147	0.029
Relationship with patients	−0.035	0.012	−0.120	−2.290	0.019
MOS-SSS	0.118	0.058	0.134	2.517	0.013
Patients					
Gender	−2.431	0.802	−0.161	−3.017	0.002
Age	−2.679	0.839	−0.173	−3.202	0.001
MBI	1.149	0.439	0.139	2.709	0.005
IADL	−0.771	0.357	−0.120	−2.152	0.023
MRMI	−0.834	0.303	−0.169	−3.266	0.001
SAS	2.148	0.611	0.233	3.603	0.000
SDS	−3.764	0.740	−0.399	−5.120	0.000
NHISS	0.335	0.097	0.357	3.441	0.001

**Table 11:** Multivariate stepwise regression analysis of factors influencing caregiver medical-social support scores.

Influence factors	Regression coefficient	Standard error	Standard regression coefficient	*T* value	*p* value
Informal caregivers					
Age	2.088	0.373	0.285	5.453	0.000
Occupational status	−0.839	0.301	−0.171	−3.267	0.001
Education degree	2.158	0.600	0.235	3.605	0.000
Relationship with patients	0.095	0.045	0.120	2.096	0.037
MOS-SSS	−0.085	0.039	−0.119	−2.173	0.030
Patients					
Gender	0.101	0.038	0.157	2.763	0.005
Age	0.080	0.037	0.115	2.061	0.039
MBI	0.105	0.050	0.126	2.258	0.024
IADL	−0.391	0.111	−0.372	−3.556	0.000
MRMI	0.223	0.092	0.234	2.461	0.015
SAS	0.265	0.123	0.198	2.203	0.027
SDS	1.150	0.445	0.145	2.710	0.006
NHISS	−0.768	0.358	−0.113	−2.153	0.033

## 4. Discussion

In this study, the informal caregivers had the highest scores in the acute stage of SAS and SDS and gradually decreased over time. This might be related to the caregivers’ lack of knowledge of the disease and treatment plan and unfamiliarity with the hospital environment. With the integration of rehabilitation training, nursing, and the country’s vigorous community rehabilitation and people’s attention to health, patients could receive rehabilitation training or self-exercise at home even after discharge, which gradually improves the patient’s functional status. The caregiver’s emotion also changed. Therefore, nursing staff should explain disease-related knowledge to caregivers early in the hospital, introduce the ward environment, gain their trust, and relieve anxiety and depression. Rehabilitation training should also be maintained until the patient is discharged from the hospital and, if necessary, establish a comprehensive evaluation of the interventions received after discharge. In addition, the burden and stress of caregivers are the most severe in the acute phase, which is gradually relieved with the extension of time, and social support is also increased. Patients with acute stroke tend to go to high-level hospitals in the expectation of better medical care, which leads to higher hospitalization costs, lower reimbursement rates and a higher burden of disease. Caregivers are also busy caring for patients, have less interaction with the outside world, and have lower scores for social support. After entering the convalescence period, the patient’s condition gradually stabilizes, the medical expenses to be paid are relatively reduced, and the sense of burden is reduced. As caregivers get used to caring for patients, they can manage their time better, and with more friends to visit, they feel more social support. Therefore, it is worth studying how to alleviate the pressure and burden of caregivers and increase social support, such as establishing and improving the hierarchical diagnosis and treatment system and two-way referral system, reducing medical costs, encouraging caregivers to seek help from others, exchanging care experience and sharing care experience with other caregivers.

The study showed that the perceived stress of caregivers was related to their occupational status, relationship with patients, and medical-social support. This is consistent with previous research results (37). In addition to fulfilling their duties, the in-service caregivers also have to take care of patients and other family members, as well as take care of household chores, so they are under great pressure. The relationship between the caregiver and the patient also affects caregiver stress, which is significantly greater for the spouse of the patient than for the nonspouse. It may be that spouses play multiple roles in the family, and any physical problems of one partner can cause significant mental and psychological problems for the other. The less social support the caregivers of stroke patients receive and the fewer resources they can use, the more powerless they will feel, the more pressure they have to take care of them, and the more likely they will have negative emotions such as anxiety and depression. Therefore, it is suggested to establish caregiver-related welfare policies, such as paid personal leave and caregiver tax exemptions, to reduce caregivers’ negative emotions by reducing their financial burden and increasing caregiving time. As well as establishing care centers, such as disease knowledge, care methods, communication between caregivers, and psychological guidance, to provide caregivers with disease-related care information and positive emotional support so that they can access ways to adjust to stress. In addition, the WeChat group, QQ and other online communication platforms can be established to strengthen communication between caregivers and alleviate their pressure through peer education.

The patient’s condition also affected the stress level of caregivers. The high disability rate of stroke leads to the loss of daily life ability of the patients and the inability to carry out normal life, which places a serious economic burden on the family. At the same time, to take care of patients, caregivers also have to invest more time and energy. Moreover, stroke recovery is a long process, and long-term care will cause serious stress to caregivers. The more severe the illness, the more care needed, and the greater the stress on the caregiver. Therefore, new medical technologies should be actively improved and innovated in clinical work, and health education of caregivers’ care skills should be strengthened to speed up patients’ recovery to promote the relief of caregivers’ pressure.

Our study found that the medical-social support situation of informal caregivers in China is pessimistic. According to the results, the MOS-SSS score of the caregivers was the lowest at the time of stroke onset, at 69.09 ± 11.13 points. As the stroke progressed, the score showed an upward trend, with the highest score of 75.76 ± 13.14, which was higher than similar studies (38) and even much higher than the normal standard. The reason may be that when the patient is newly ill, the caregiver needs to invest much more energy to deal with various situations of suddenness and has less contact with the outside world than before. Therefore, in this stage, social support was the lowest. As the condition is stable, the care, help and encouragement of colleagues, relatives and friends from the workplace and the health education of medical staff provide support the caregiver more. After returning to the family, social organizations such as community hospitals and nursing homes can also provide social support to them. Therefore, in future studies, we might strengthen the health education of care, attach much importance to the development and maintenance of social support networks, and help them improve their ability to establish and utilize social support networks to improve social support.

## 5. Conclusion and limitations

Stroke patients need high quality of care due to long-term dysfunction. In most countries, care is carried out by informal caregivers. However, not only informal caregivers have no work remuneration, but they also have to pay for providing care. In particular, it takes time and leads to mental stress and physical exhaustion for caregivers, which then affect their occupation and health. Only when medical professionals are more aware of the basic characteristics and values of informal caregivers and more comprehensively understand them from the physical, psychological and social aspects can we better develop relevant interventions and improve their health. This also provides a better guarantee for patients’ health.

This study is more concerned with the psychological status of informal caregivers. Future research should include a comprehensive, long-term understanding and observation of the physical, psychological and sociological status. Meanwhile, because this study is only for caregivers of normal cognitive stroke patients, in future studies, the entire stroke group can be included. In addition, this study only included patients within 72 h, 2 months and 3 years after stroke onset and cannot fully represent the pressure and mental state of informal caregivers in different periods of stroke patients. In addition, studies have shown that caregivers’ stress is negatively correlated with patient cognitive function, activities of daily living, and ADL scores (39). Patients included in this study were all stroke patients with normal cognitive function; therefore, their daily living activities and functional status were better than those of the entire stroke group, which also affected the emotion and stress of the caregiver to some extent.

## Data availability statement

The raw data supporting the conclusions of this article will be made available by the authors, without undue reservation.

## Ethics statement

The studies involving human participants were reviewed and approved by West China Hospital of Sichuan University Biomedical Research Ethics Committee. The patients/participants provided their written informed consent to participate in this study.

## Author contributions

NH designed this study, and ZL is the guarantor for the article. YT and PZ collected data. NH and XG analyzed the data. NH, YT, PZ, and XG drafted the paper, which was revised by ZL. ZL will serve as an adviser for methodology. The study’s publishing has been authorized by all of the authors.

## Funding

This study was supported by the Sichan Association for Science and Technology (2020YFS0169).

## Conflict of interest

The authors declare that the research was conducted in the absence of any commercial or financial relationships that could be construed as a potential conflict of interest.

## Publisher’s note

All claims expressed in this article are solely those of the authors and do not necessarily represent those of their affiliated organizations, or those of the publisher, the editors and the reviewers. Any product that may be evaluated in this article, or claim that may be made by its manufacturer, is not guaranteed or endorsed by the publisher.
